# Deep Genealogy and the Dilution of Risk

**DOI:** 10.1371/journal.pbio.1001660

**Published:** 2013-09-17

**Authors:** Roland G. Roberts

**Affiliations:** Public Library of Science, Cambridge, United Kingdom

We now know that many of the common diseases that afflict human populations are at least partly caused by the genes we inherit. In contrast to the classic rare “Mendelian” genetic diseases, where inheritance of rare but severe mutations at a single gene can make the difference between sickness and health, the genetic risk of getting common maladies like, for example, Alzheimer disease, diabetes, or depression is altogether fuzzier in nature. Here, common and individually mild mutations in many genes combine to raise our personal susceptibility to the point at which they might connive with environmental factors to trigger the disease state.

**Figure:**
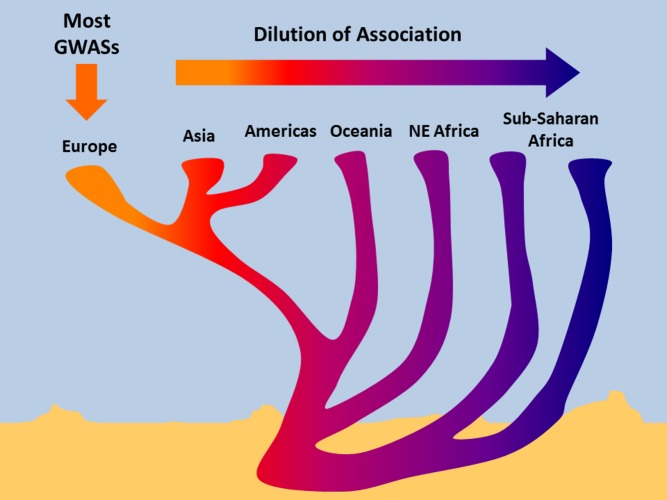
Most genetic studies of common diseases have been performed in populations with European ancestry; the genetic effects that they identify are progressively diluted in people from other continents.

It's obviously much harder to pin down this distributed kind of genetic risk than it is to identify the mutations responsible for a Mendelian disease, but the last decade and a half has seen the rise of a clever solution—the Genome-Wide Association Study or GWAS, a triumph of technology, statistics, and sheer ambition. To do a typical case-control GWAS you first need blood samples from hundreds or thousands of individuals, divided into two groups—one with and one without a particular disease or trait. You then need to check the genotype—that is, the genetic variants—at hundreds of thousands of sites across the genome in each sample (these variable sites are called single nucleotide polymorphisms or SNPs). Finally you use some sophisticated statistical tools to see whether a given SNP is “associated” with the disease state, i.e., whether one variant is significantly more common in people who have the disease than in those who don't. Typically this association is subtle, with most GWAS “hits” affecting risk by less than a factor of two. A significant association between a SNP and a disease indicates that some other variant near the SNP in the genome (the “functional variant”) directly affects a person's biological function in a way that raises or lowers their risk of that disease.

This brings us to an uncomfortable truth. It's clear from the above that to even attempt a GWAS you need a lot of money, some serious kit, and a robust medical infrastructure. Presumably as a result of this, the vast majority of GWASs have been done on individuals of European ancestry, mostly in Western Europe and Northern America. However, there are seven billion of us on this planet, and most of us don't share this ancestry. To what extent can we simply extrapolate the results of these hundreds of GWASs to the rest of the world's population?

This is the question directly posed in a *PLOS Biology* paper by Christopher Carlson, Charles Kooperberg, and colleagues. They used DNA samples from more than 73,000 people describing themselves as having a range of ancestries (50% European, 20% African American, 11% Hispanic, 8% Native American, and so on). For each of the 73,000 individuals the authors then checked the genotype of 68 SNPs known (from studies of Europeans) to be associated with body mass index (BMI), type 2 diabetes, or levels of fats in the bloodstream.

To ensure that they were making a fair comparison, the authors first checked that these previously reported associations are seen in their own European cohort. They then assessed the so called direction (see below) of effect of each SNP in individuals of non-European ancestry. For almost all associations between a SNP and a trait, the effect *direction* is the same in other populations; thus a genotype that's associated with increased risk of a disease (or related trait) in Europeans will also tend to be associated with an increased (rather than decreased) risk in people from other ancestries. However, this study found that the *size* of the effect differed substantially between continental populations, with the most profound effects seen in African Americans, where a quarter of the associations were appreciably weaker, often to the point of disappearing altogether (see Figure).

What's the reason for this? Remember that when we're studying genetic associations, what we're usually looking at is an intrinsically innocuous SNP that's merely a proxy for the functional variant. And although these genetic loci are physically close to each other in the genome, the genetic shuffling or recombination that occurs in each generation can, over time, separate them from each other. There's also the possibility that different functional variants exist in different populations, and that functional variants depend on other genetic or environmental factors (which might in turn differ between populations) to have their effect.

The authors then used some additional high-resolution genetic data on their European and African American populations to distinguish between these possibilities, and they find that much of the dilution is due to the genetic scrambling that happens as SNPs and causal variants are passed from parents to offspring; the more generations that separate two individuals from each other, the worse the SNP is at tagging the functional variant. Indeed, in half of those diluted associations, the authors see evidence that African populations nevertheless have the same underlying functional variant, with the same strength of association, but tagged by a different SNP.

Few will be surprised by these results, but this study does several important things. First (the bad news), it clinches and quantifies the problem of extending current genetic risk models, based on associations from European populations, to humans whose origins lie in other continents. Second (the good news), it suggests that in a future sun-blessed time when we've identified all the functional variants (a non-trivial achievement)—rather than their potentially deceptive SNP stand-ins—genetic models *will* be more readily transferrable across all of humanity. Third, it makes a strong and specific case for carrying out genetic association studies in non-European populations, with those of African ancestry being the single most beneficial target.

**Carlson CS, Matise TC, North KE, Haiman CA, Fesinmeyer MD, et al. (2013) Generalization and Dilution of Association Results from European GWAS in Populations of Non-European Ancestry: The PAGE Study. http://dx.doi.org/10.1371/journal.pbio.1001661**

